# Integration of High-Performance InGaAs/GaN Photodetectors by Direct Bonding via Micro-transfer Printing

**DOI:** 10.1021/acsami.3c17663

**Published:** 2024-02-13

**Authors:** Yang Liu, Zhi Li, Fatih Bilge Atar, Hemalatha Muthuganesan, Brian Corbett, Lai Wang

**Affiliations:** † Beijing National Research Center for Information Science and Technology (BNRist), Department of Electronic Engineering, Tsinghua University, Beijing 100084, China; ‡ Tyndall National Institute, University College Cork, Cork T12 K8AF, Ireland

**Keywords:** micro-transfer printing, direct bonding, van der Waals force, interface states, interface processing, photodetectors

## Abstract

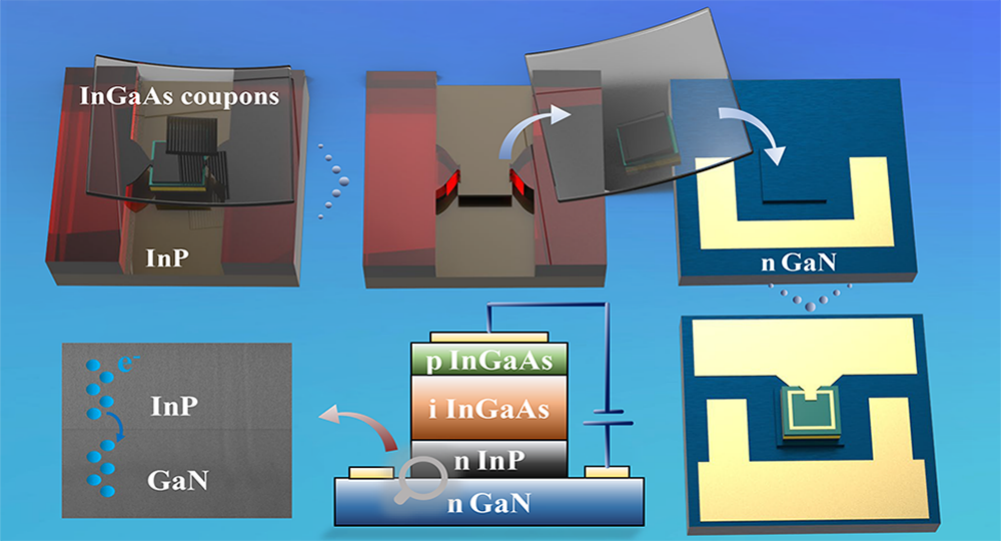

The integration of dissimilar semiconductor materials holds immense potential for harnessing their complementary properties in novel applications. However, achieving such combinations through conventional heteroepitaxy or wafer bonding techniques presents significant challenges. In this research, we present a novel approach involving the direct bonding of InGaAs-based p-i-n membranes with GaN, facilitated by van der Waals forces and microtransfer printing technology. The resulting n-InP/n-GaN heterojunction was rigorously characterized through electrical measurements, with a comprehensive investigation into the impact of various surface treatments on device performance. The obtained InGaAs/GaN photodetector demonstrates remarkable electrical properties and exhibits a high optical responsivity of 0.5 A/W at the critical wavelength of 1550 nm wavelength. This pioneering work underscores the viability of microtransfer printing technology in realizing large lattice-mismatched heterojunction devices, thus expanding the horizons of semiconductor device applications.

## Introduction

The integration of semiconductor materials across divergent systems poses a formidable challenge, characterized by inherent disparities encompassing lattice constant mismatch, distinct growth conditions, phase separation dynamics, and so on.^1−7^ However, it has been proven that one could benefit from such combinations by exploiting the advantages of different materials and exploring a variety of new applications.^8−11^ For instance, through integration, III–V optoelectronic devices can be incorporated into silicon-based chips, thus enabling the utilization of silicon in domains such as optical communication and optical computing. Furthermore, the integration of materials boasting diverse bandgaps enables the enhanced absorption of a broader spectrum of light, translating into superior photovoltaic conversion efficiency.^12−14^

The III–As materials are very suitable for devices operating with wavelengths in the data communication band (1300–1600 nm), with superior crystal quality due to a mature growth process.^15−17^ On the other hand, III–N materials usually have high electron mobility and excellent thermal conductivity, which is suitable for power electronic devices.^18−20^ Therefore, combining both material systems could benefit high-frequency and high-power applications in the field of optical fiber communications. Wafer bonding has been used to form the large lattice-mismatched heterojunction, such as GaAs and GaN. However, a central challenge resides in the stringent prerequisites imposed by direct wafer bonding, necessitating exceedingly low surface roughness on both materials and an exceptionally clean bonding environment.^21−23^ Elevated surface roughness undermines the effective contact area between the wafers and, concurrently, amplifies the surface energy of the wafers, thereby adversely impacting bonding strength.^24−26^ Additionally, disparate thermal expansion coefficients between the two materials render them susceptible to detachment under external mechanical forces or within thermally dynamic environments.^27,28^

Recently, micro-transfer printing (MTP) technology has been introduced for the integration of electronic and optoelectronic devices, such as silicon-based optoelectronic integration and flexible electronic devices.^29−33^ By integrating disparate semiconductor devices onto a unified substrate, MTP technology enhances the overall efficiency and compactness of integrated systems, while concurrently streamlining the intricacies associated with packaging processes.^34,35^ However, achieving high-quality bonding through van der Waals forces and optimizing the interfacial properties of the heterojunction is worthy of in-depth study.^36−38^ Furthermore, the forthcoming challenges encompassing device reliability and durability after bonding and the development of robust characterization and testing methodologies will need to be addressed in the future.

In this paper, we released the InGaAs (PIN)/InP membranes from the original InP substrate and then directly transfer-printed the devices to n-GaN through MTP technology, to realize a heterojunction of large lattice mismatch and demonstrate the carrier transport between the two materials. The comprehensive characterization of the resulting n-InP/n-GaN interface encompassed cross-sectional scanning electron microscopy, voltage–current measurements, and Kelvin probe force microscopy (KPFM) assessments. Additionally, our investigation incorporated the systematic application of various surface treatments to elucidate their effects on interface properties. Furthermore, we conducted an in-depth analysis of the electric and optical properties exhibited by the InGaAs/GaN photodetector. Notably, this device showcased highly promising electrical characteristics and demonstrated remarkable optical responsivity, specifically recording a peak responsivity of 456 mA/W within the 1550 nm wavelength range. These findings underscore the significance of our research in advancing the fields of semiconductor integration and heterojunction development.

## Experimental Details

**Figure 1:**
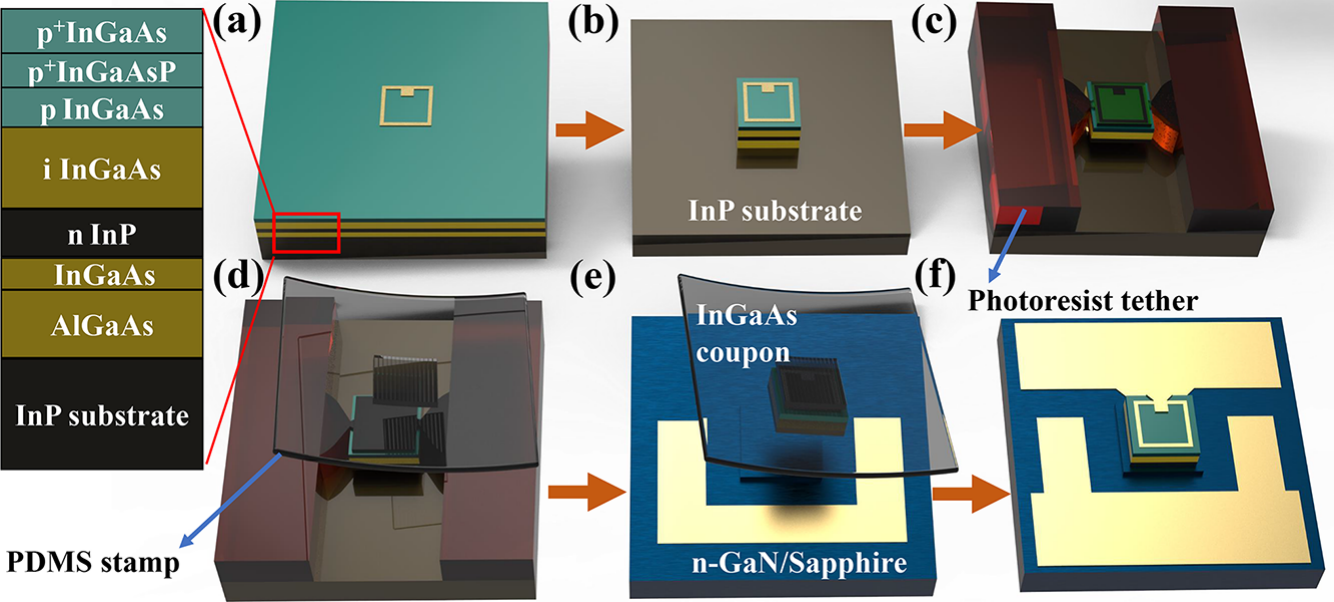
(a) Evaporate the Ti/Pt/Au electrode of p-InGaAs; (b) ICP etch to form InGaAs mesa; (c) lithography to form tether and undercut the InGaAs/AlGaAs release layer; (d) use PDMS stamp to pick up the InGaAs coupon; (e) transfer print the InGaAs coupon to the middle of n-GaN mesa; and (f) the detector was fabricated after standard fabrication process.

**Figure 2:**
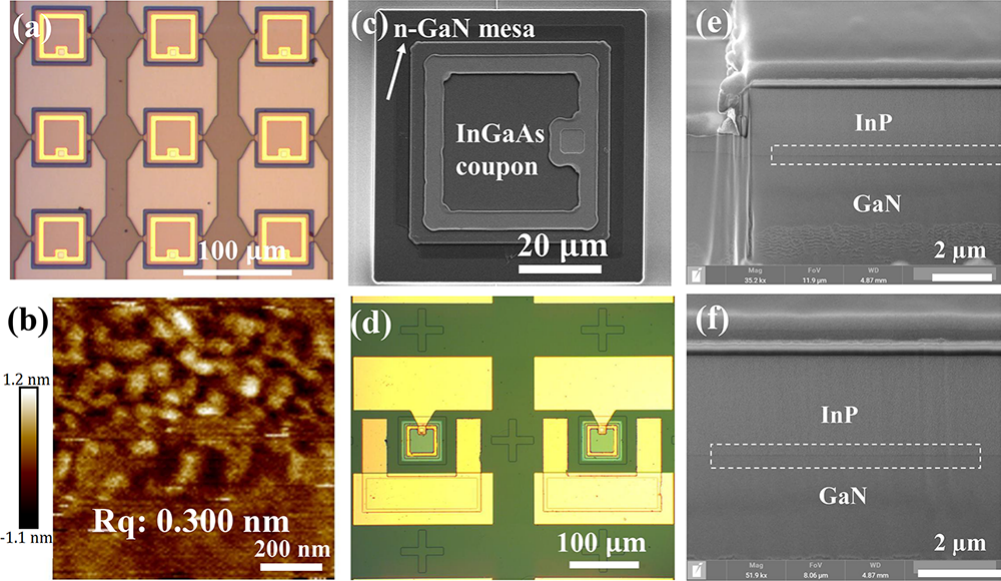
(a) Optical image of InGaAs coupons fixed by photoresist tether; (b) AFM image (1 μm × 1 μm) of the lower surface of InGaAs coupons after wet etching; (c) SEM image of InGaAs coupon transfer-printed on the middle of GaN mesa; (d) optical image of the InGaAs/GaN detector after fabrication process; (e) SEM image of the InP/GaN interface captured from the edge of the coupon using FIB; and (f) SEM image of the InP/GaN interface captured from the center of the coupon using FIB.

The PIN InGaAs membrane is derived from the InP epitaxial wafer (as shown in Figure 1), which is comprised of 275 nm-thick p-type InGaAs(P), 250 nm-thick intrinsic InGaAs, 150 nm-thick n-type InP, and the sacrificial layers consisting of InGaAs (400 nm)/AlGaAs (100 nm). The schematic of the whole process is shown in Figure 1. To prepare the PIN membranes (or “coupons”) for transfer printing, a metal contact using Ti/Pt/Au was first formed on top of p-InGaAs by standard lift-off process. Then 50 μm × 50 μm mesas were formed using the inductively coupled plasma (ICP) etching to etch until the n-InP layer. The top InGaAs layers were then covered by a SiO_2_ protective layer to prevent corrosion from the etchant solution, followed by the second ICP etch using SiO_2_ as hard masks to expose the bottom InGaAs/AlGaAs release layers. A photoresist layer is subsequently coated and patterned by the photolithography technique and is used as the tether to support the coupons during the releasing process. FeCl_3_:H_2_O (1:2) was used to selectively wet etch InGaAs/AlGaAs layers and extract the top InGaAs/InP membranes. The optical image showing the completely released InGaAs/InP coupons anchored by the photoresist tethers on the wafer surface is shown in Figure 2a.

Meanwhile, on the n-GaN/sapphire wafer, 68 μm × 68 μm n-GaN mesas were formed by ICP etching, and a Ti/Au contact was then evaporated around the mesas to form an ohmic contact. Once the InGaAs/InP release was completed, the PIN coupons with electrodes were directly transfer-printed onto the pretreated n-GaN mesa surfaces, without using any adhesion layer. Due to the less stringent requirements for direct bonding between such nanomembranes and wafers compared to direct bonding between wafers, the success rate of transfer printing is relatively high, and it yields a high bonding quality. The bonded structure can withstand subsequent processes, such as photolithography and cleaning. Finally, the whole structure was passivated by the SiO_2_ layer, and a metal bond pad connecting the p-metal of the printed coupons was deposited.

To investigate the properties of the InP/GaN interface, coupons with only the n-InP layer were also released and printed onto the n-GaN surface. Before commencing the transfer printing process, a comprehensive array of surface treatments was carried out on the n-GaN substrate, including (1) surface cleaning with H_2_SO_4_:H_2_O_2_ = 3:1 for 5 min and buffered oxide etch (BOE) for 5 min (labeled as “standard cleaning”); (2) standard cleaning followed by a 30 s immersion in 45 wt % KOH aqueous solution at 100 °C (labeled as “KOH”); (3) standard cleaning followed by oxygen plasma treatment for 5 min (labeled as “O_2_ plasmas”); and (4) standard cleaning followed by the coating of a monolayer of hexamethyldisilazane (HMDS) onto the GaN surface (labeled as “HMDS”). During the transfer printing process, variations in surface treatments did not show any significant impact on the success rate of the transfers. After transfer printing of n-InP on the n-GaN targets, the heterojunction was annealed at 420 °C in ambient N_2_ to observe the change of the interface characteristics before and after annealing.

To investigate the heterojunction characteristics, two probes were applied onto both contacts (one is on p-AlGaAs or n-InP and another one on n-GaN), and current–voltage (*I*–*V*) curves were recorded. To check the roughness on the etched side of the released coupons, a bulk PDMS stamp was used to pick up the coupons, and the atomic force microscope (AFM) (Bruker Dimension Icon) was used to measure the root-mean-square (RMS) roughness. To examine the bonding interface as well as the surface morphology, focused ion beam scanning electron microscopy (FIB-SEM) (Tescan Solaris) and field emission SEM (QUANTA 650 HRSEM) were used. To measure the optical response, an infrared testing system based on a 1550 nm tunable laser was used. In the experiment, a Keithley 2400 tester is used to test the electrical performance of the device. Agilent 8164B lightwave measurement system was used to test the optical response characteristics of devices at 1550 nm wavelength light.

## Results and Discussion

Figure 2b shows the AFM result from the 1 μm × 1 μm area of the released InGaAs/InP backside surface after wet etch. The low RMS roughness value (i.e., 0.3 nm) is attributed to the highly selective undercut etching process using the FeCl_3_:H_2_O solution. It is imperative to emphasize the critical significance of a smooth backside when employing transfer printing technology for direct bonding. This smooth backside condition is pivotal as it ensures the feasibility of directly printing coupons onto a pristine surface and subsequently bonding them via van der Waals forces, obviating the need for additional adhesive layers. As illustrated in Figure 2e, the smooth backside surfaces facilitate the formation of an intimate atomic-level contact at the InP and GaN heterojunction. This level of contact is conducive to the unhindered passage of carriers through the interface, thereby enhancing the overall functionality of the semiconductor device.

As shown in Figure 2c, the PIN coupons were successfully printed on n-GaN mesas. The printed InGaAs-based membrane exhibited intact structures and a flat surface without any mechanical damage. The interface was further examined by FIB cuts on the edge and center parts of the printed coupons, respectively (as shown in Figure 2e). Both bonded areas exhibit a sharp interface without any voids or gaps, indicating close contact between InP and GaN layers. It is believed that the successful direct bonding process through transfer printing is mainly attributed to the smooth surfaces on both the InGaAs/InP backside and the n-GaN surface. Poor surface morphology or smoothness would result in the failure of the printing process.

**Figure 3:**
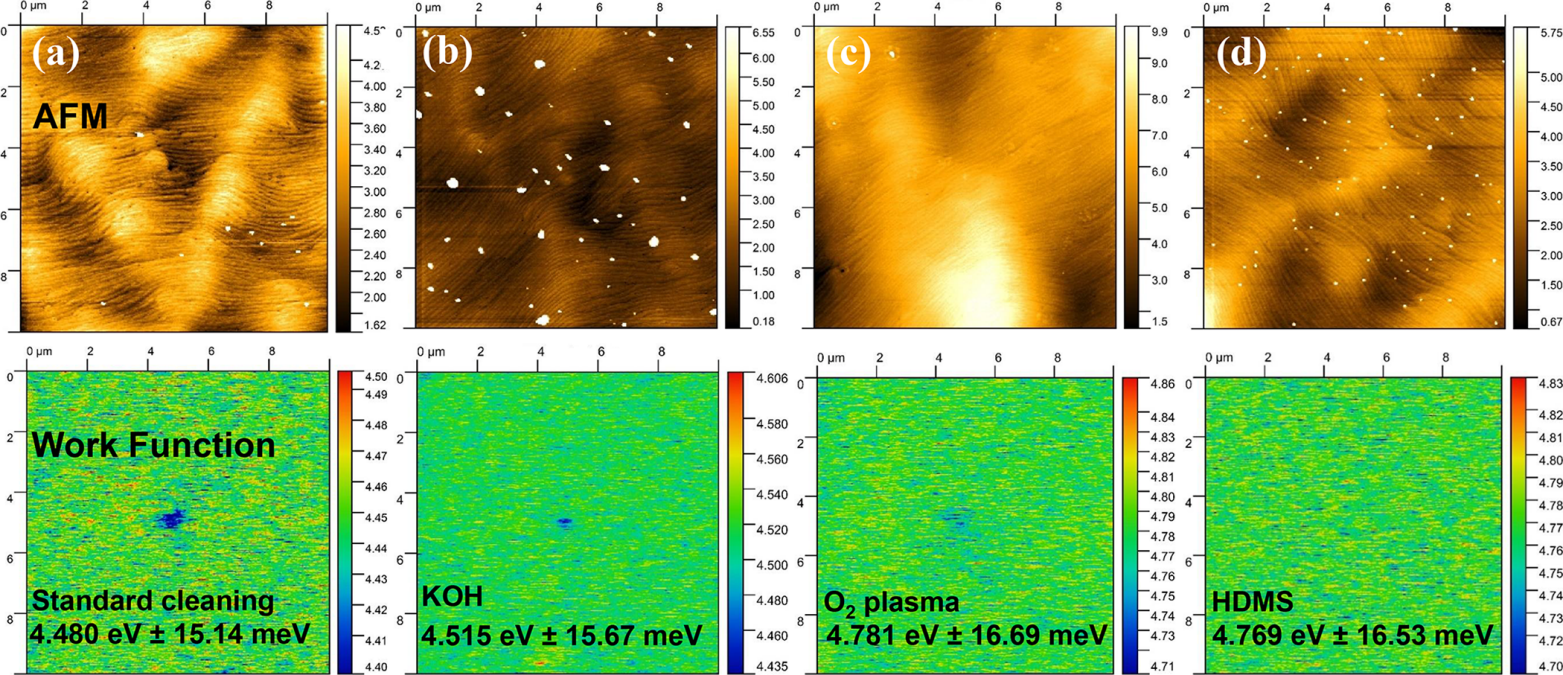
KPFM images illustrating various surface treatments applied to the n-GaN layer: (a) standard cleaning process; (b) KOH treatment; (c) O_2_ plasma treatment; and (d) HDMS treatment.

To investigate the characteristics of the n-InP/n-GaN interface, which is the most important junction in the InGaAs/GaN PIN detector, KPFM was used to get the work function of the n-GaN surface under different kinds of treatment. The work function of n-GaN affects the barrier height at the interface. As shown in Figure 3, the surface work functions of n-GaN with standard cleaning and with KOH treatment are similar, with the values around 4.5 eV, which is just above the theoretical value of electron affinity (i.e., 4.1 eV). This may be due to the fact that the surface of n-GaN becomes relatively fresh after standard cleaning, and further KOH cleaning does not modify it much. The work function increases when the O_2_ plasma is applied. The O_2_ plasma bombards the surface, causing the surface to form a recombination center, and some electrons are bound, resulting in a decrease in the Fermi level of n-GaN and an increase in the work function. For HMDS, organic materials often have polar molecules or functional groups with dipoles (regions of positive and negative charge) present in them. When these organic molecules adsorb onto the semiconductor surface, the dipoles can interact with the semiconductor atoms or surface states. This interaction can result in the formation of a surface dipole layer, which causes the increase of work function of n-GaN.

**Figure 4:**
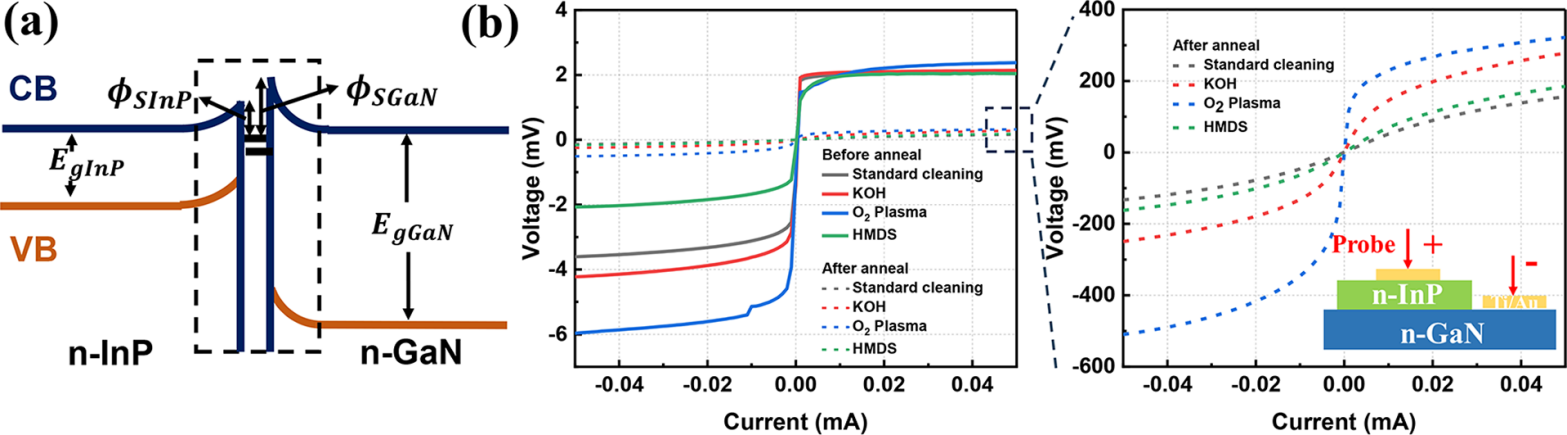
(a) Schematic diagram of electron affinity of InP and GaN, and energy band diagram of the n-InP/n-GaN junction; (b) VI characteristic of the n-InP/n-GaN junction with different surface treatments before and after annealing.

The *V*–*I* characteristics of the n-InP/n-GaN heterojunctions with various treatments were measured before and after annealing, with the results shown in Figure 4. Note that in these tests, the positive voltage is applied to n-InP electrodes, while the negative voltage is applied to n-GaN electrodes. Before annealing, all devices show rectifying characteristics, regardless of the surface treatments, indicating a Schottky barrier at the n-InP/n-GaN interface. This can be explained by the energy band diagram of the heterojunction at thermal equilibrium, as shown in Figure 4a. GaN exhibits a wurtzite crystal structure with a polarized electric field in the *c*-axis direction due to the fact that the centers of positive and negative charges do not coincide along the longitudinal axis. For the Ga face of GaN material, the spontaneous electric field direction extends from the interior toward the surface, leading to an upward curvature of the surface’s energy band. Simultaneously, with regard to InP material, at the surface of InP, periodic lattice structure termination gives rise to surface states. These surface states induce defect energy levels within the bandgap, thereby immobilizing free electrons and creating a pinning effect on the Fermi level within the bandgap of n-InP at the surface.^39,40^ Consequently, at the interface of the heterojunction, as the Fermi level flattens, both the conduction and valence bands on either side experience an upward shift relative to that of the Fermi level. Thus, at the interface, as electrons transit from n-InP to n-GaN, they encounter potential barriers formed by the band offsets and band bending.

As Figure 4a illustrates, due to the curvature of the energy bands on both sides, the depletion layers form on either side of the interface. The interface can be regarded as a quasimetallic layer, allowing one to conceptualize the heterojunction diode as a combination of two Schottky junctions. Therefore, this n-InP/n-GaN heterojunction can be viewed as a series connection of the n-InP Schottky junction and the n-GaN Schottky junction.

Although all devices exhibit the same level of voltage at the forward bias, the reverse voltages are quite diverse. For instance, subsequent treatment with oxygen plasma results in an elevation of the device’s reverse voltage. This phenomenon may be attributed to the effect of O_2_ plasma bombardment on the GaN surface, leading to an increased density of interface states, which also causes the increase of the GaN work function. Conversely, the device coated with HMDS demonstrates the lowest reverse voltage, and its current–voltage curve manifests symmetry with respect to the origin. Upon HMDS adsorption onto the n-GaN surface, the associated dipoles engage with surface states, thereby engendering a dipole layer that imparts modifications to the energy levels at the interface. Through this energy level adjustment, the HMDS assists carriers in surmounting energy barriers at the interface.

After annealing the devices, the on resistance of the devices reduced and changed to quasi-linear behaviors, indicating the ohmic contacts formed. Our preliminary experiments have demonstrated that annealing has a much weaker effect on the n-electrode contact compared with its impact on the heterojunction interface. On one hand, it is likely that during the annealing process at 420 °C, some out-diffusion process of P atoms from the InP layers happens, leading to the formation of P vacancies acting as donors. Therefore, a highly doped layer is formed at the interface, which eventually lowers the barrier height eventually. On the other hand, it is also possible that annealing improves the contact between the two materials in the heterojunction, which helps reduce the potential barrier at the interface.

The heterojunctions that underwent the KOH treatment exhibited subtle variations. Devices that did not undergo KOH treatment displayed lower reverse bias voltages, lower ON resistances, and more linear quasi-Ohmic contact IV curves. This suggests that mild oxide formation on the GaN surface postannealing led to the recrystallization of the interface, resulting in a tighter connection of the heterojunction. This passivated surface states, reduced interface defect density, and endowed the heterojunction with quasi-Ohmic contact characteristics. In contrast, devices subjected to oxygen plasma treatment exhibited the least improvement in Ohmic contact quality due to surface damage caused by the plasma. The possible reason is that 420 °C is not enough to repair the surface damage caused by O_2_ plasma. Simultaneously, devices treated with HMDS displayed excellent quasi-Ohmic contact characteristics after annealing.

**Figure 5:**
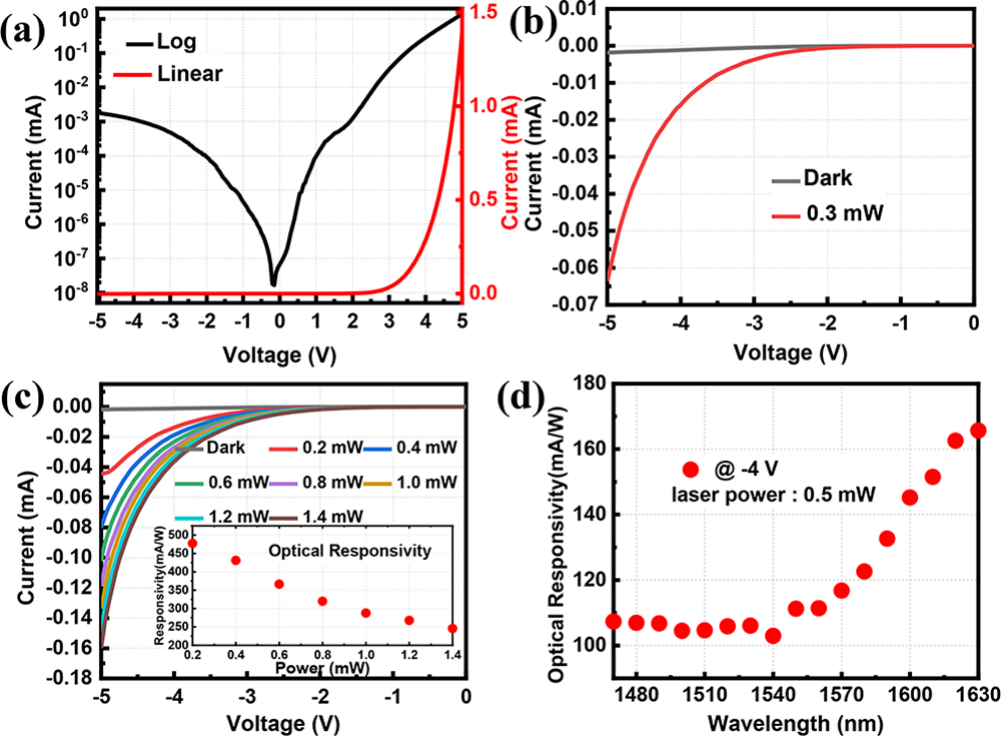
(a) Linear and semilogarithmic plot of the IV characteristic curve of InGaAs/GaN detector at room temperature; (b) photoresponse characteristics from 0 to −5 V under 1550 nm laser light; (c) photoresponse characteristics with different laser intensities, the inset is the corresponding photoresponsivity under different light intensity; and (d) photoresponse characteristics with different wavelength of laser.

Figure 5a presents the electrical performance of the InGaAs/InP/GaN PIN detector fabricated based on the critical heterojunction InP/GaN. As depicted in the figure, this device exhibits typical electrical characteristics of a PIN-type device. Due to the bandgap mismatch between p-type InGaAs and n-type GaN at the device’s two ends, an internal electric field is established within the device, directed from GaN toward InGaAs. Additionally, the InP/GaN heterojunction already formed a well-defined quasi-Ohmic contact. Consequently, the influence of this InP/GaN heterojunction on the internal electric field of the overall PIN structure is relatively minimal. This attribute imparts rectifying characteristics to the device. As shown in Figure 5a, the heterojunction detector exhibits diode characteristics in the range of ±5 V.

To assess the detector’s response to 1550 nm wavelength light, a 1550 nm laser was employed. Given that GaN does not efficiently absorb light at this wavelength, only 46.5% of the incident laser light is absorbed by the device. This is illustrated in Figure 5b, where, at an incident light intensity of 0.3 mW, the InGaAs/GaN detector exhibits a photoresponsivity of 456.2 mA/W. This observation underscores the capacity of the InGaAs absorption layer to absorb photons, generating photoinduced carriers. These carriers can subsequently traverse the InP/GaN heterojunction interface, culminating in the formation of a photocurrent within the device. Consequently, GaN-based devices exhibit sensitivity to light at a wavelength of 1550 nm.

As shown in Figure 5c, the optical response curves of the detectors change under different laser intensities at a 1550 nm wavelength. The inset shows the corresponding photoresponsivity under different light intensity. With the increase of light intensity, the light response gradually tends to saturation. The generation of electron–hole pairs reaches a point where the available recombination centers become saturated. As more and more carriers are generated, a significant portion must recombine before they can contribute to the electrical current. At the same time, Figure 5d shows the relationship between photoresponsivity and the wavelength of incident laser ranging from 1470 to 1630 nm. The response curve matches the InGaAs absorption curve at the wavelength of 1550 nm.^41^ This congruence signifies the efficacy of the detector in harnessing incident photons at this specific wavelength, substantiating the compatibility between the device’s responsivity profile and the spectral characteristics of the InGaAs absorption band. It demonstrates the successful integration of InGaAs detector attributes onto a GaN-based platform, effectively overcoming the inherent bandgap width constraints of the GaN material system. Consequently, this advancement extends the detector’s responsiveness into the crucial communication band, opening up new possibilities for infrared detection applications. It is worth noting that the bonding associated with transfer printing demonstrated in this work is highly reproducible, and one can expect that the larger-scale production of various heterogeneous devices can be achieved. However, further investigations such as precise control of the undercut etching process, uniformity control across large wafer surfaces, and printing optimization using large, arrayed stamps will be required.

## Conclusions

In summary, this study leveraged MTP technology to achieve direct bonding of InGaAs-based p-i-n membranes on GaN surface, effectively addressing challenges arising from lattice mismatch. A flat and sharp bonding interface was achieved at the n-InP/n-GaN layers, resulting from the atomically smooth surfaces of InP after release etching. Investigations on the influence of wet treatments showed that the treatment modifies the surface work function of n-GaN, leading to different carrier transport behaviors for n-InP and n-GaN heterojunctions at the reverse bias. Ohmic (or quasi-Ohmic) contact was achieved after the heterojunctions. InGaAs/InP/GaN PIN detector with an optical responsivity of 456 mA/W at 1550 nm wavelength was demonstrated. The integration of InGaAs detector capabilities onto a GaN-based platform represents a breakthrough, surpassing limitations imposed by the band gap in the GaN material system. This achievement significantly extends the device’s response range into the communication band, holding great promise for applications in infrared detection. Future research may focus on further optimizing interfacial properties and exploring novel material combinations for even more versatile semiconductor integration.
